# Toward a standard formal semantic representation of the model card report

**DOI:** 10.1186/s12859-022-04797-6

**Published:** 2022-07-14

**Authors:** Muhammad Tuan Amith, Licong Cui, Degui Zhi, Kirk Roberts, Xiaoqian Jiang, Fang Li, Evan Yu, Cui Tao

**Affiliations:** School of Biomedical Informatics, University of Texas Health Science Center, Houston, TX USA

**Keywords:** Knowledge representation, Ontology, Model cards, FAIR, Standardization, Semantic web, Machine learning, Artificial intelligence

## Abstract

**Background:**

Model card reports aim to provide informative and transparent description of machine learning models to stakeholders. This report document is of interest to the National Institutes of Health’s Bridge2AI initiative to address the FAIR challenges with artificial intelligence-based machine learning models for biomedical research. We present our early undertaking in developing an ontology for capturing the conceptual-level information embedded in model card reports.

**Results:**

Sourcing from existing ontologies and developing the core framework, we generated the Model Card Report Ontology. Our development efforts yielded an OWL2-based artifact that represents and formalizes model card report information. The current release of this ontology utilizes standard concepts and properties from OBO Foundry ontologies. Also, the software reasoner indicated no logical inconsistencies with the ontology. With sample model cards of machine learning models for bioinformatics research (HIV social networks and adverse outcome prediction for stent implantation), we showed the coverage and usefulness of our model in transforming static model card reports to a computable format for machine-based processing.

**Conclusions:**

The benefit of our work is that it utilizes expansive and standard terminologies and scientific rigor promoted by biomedical ontologists, as well as, generating an avenue to make model cards machine-readable using semantic web technology. Our future goal is to assess the veracity of our model and later expand the model to include additional concepts to address terminological gaps. We discuss tools and software that will utilize our ontology for potential application services.

## Background

The FAIR principle [1] serves as a set of guidelines for researchers and data publishers for practices involving the release of digital research assets to be *Findable* (encoded with discoverable, clear metadata for indexing), *Accessible* (persistent availability of data through unique identifiers), *Interoperable* (integration through the utilization of shared terminologies for metadata), and *Reusable* (data resources to be well-documented to be effectively integrated with other environments).

The National Institutes of Health (NIH) is invested in the use of artificial intelligence (AI) and machine learning (ML) that address the need for data and tools to unite biomedical and behavioral researchers [2]. The principles outlined by the FAIR guidelines can assist in this initiative towards tools and data that are not only FAIR, but “credible, ethical, and generalizable” to enhance scientific endeavors and enterprises [2]. Model cards are one of the resources encouraged by NIH through their Bridge2AI program [3]. Model card reports, a one to two-page document, inform researchers, software developers, stakeholders, and impacted individuals (like patients) of what a specific ML approach is capable of performing, and potential technical or ethical issues [4]. For a lay description, model card reports are akin to nutritional or drug labels mandated by the Food and Drug Administration (FDA) to inform the consumer of food and drug products.

Nonetheless, as a static document they do limit opportunities to permit the information contained within the report to be computable. If the information in the model card were processable by machines, this could allow for sophisticated opportunities such as tools and analytical tasks that can help advance FAIR guidelines. One technological approach are ontologies which are part of the technology stack of Tim Berners-Lee’s Semantic Web vision, a vision in which the web evolves from a document presentation layer to a web of linked computable electronic resources [5].

Ontologies are representational artifacts that model domain information. Within these artifacts are complex networks of concept terms that abstract information and knowledge of a specific domain. The life sciences community has been major contributors to the body of biomedical ontological resources and as evident on the Linked Open Data Cloud [6–8]. Many of these life sciences ontology resources, particularly those that are found in the Open Biological and Biomedical Ontology (OBO) Foundry, are well-documented, comprehensive knowledge bases, and have rigorous standards for design [9]. The majority of the life sciences ontologies, utilize the Basic Formal Ontology (BFO), which allows for a common framework for interoperability between various life science-related knowledge bases [10].

For background purposes, a concept is a unit of thought that binds a symbol or sign (i.e. term) with a real entity in the world. An ontology utilizes machine-level syntax to represent this concept that connects with some real and verifiable entity. These concepts are broken down into types (i.e. classes or categories) to indicate the variation of the concept, basically elucidating a taxonomy. While ontologies can be strictly a hierarchical taxonomy, some (and more semantically richer) ontologies can be polyhierarchical networks of concepts. This is accomplished through links, sometimes referred to as properties or predicates, that connect the various concepts to express some statement about the world or domain. For example, given two concepts from the Software Ontology, *algorithm* and *software*, that are linked together by a property of *implements*, the ontology representation of *software* > *implements* > *algorithm* expresses a schema elucidating some domain knowledge. Furthermore, this schema can serve as a scaffold for instance data to evoke meaning. For example, *Acme Software* is a *software*, *differential evolution (acme-variant)* is an *algorithm*, and with our sample schema, one can derive that *Acme Software* > *implements* > *differential evolution (acme-variant)*. These expressions (i.e. axioms) can be coded using OWL2 (Web Ontology Language) [11], RDF (Resource Description Framework) [12], or Turtle [13] into the ontology artifact and linked to other expressions to form a knowledge base. The logic-based consistency of the encoded expressions can be checked using a variety of available semantic reasoners, including inferring additional information. To illustrate, given *is implemented by* that is a mirrored, inverse of *implements*, an inferred axiom can be generated of *differential evolution (acme-variant)* > *is implemented by* > *Acme Software*. Lastly, the ontology can be linked to other ontologies or distributed heterogeneous resources to expand the meaning of the data. In addition, the linking of heterogeneous data resources provides functions to query the data using a query language like SPARQL [14] or SQWLR [15].

### Objective

The objective of this study is to initiate standardization of the information embedded in model card reports using formal ontology methods and resources. We present our initial work on an ontology called the Model Card Report Ontology (MCRO) to represent information and metadata from model cards in a formal conceptual structure of its report. This ontology leverages verified resources like the Basic Formal Ontology (BFO) [10], Information Artifact Ontology (IAO) [16, 17], Provenance Ontology (PROV-O) [18, 19], and the Software Ontology (SWO) [20, 21] to help enrich the core model of this ontology.

By using an ontology to construct our formal representation of model cards, we can potentially standardize the nomenclature and representation of model card reports, essentially producing a computable version of the model card that can be shared, reused, and linked. Noted earlier, this proposed ontology will utilize BFO-based ontologies that have an open-sourced, community consensus on terms and terminological structure. Utilizing these existing ontologies provides rigorous scientific standards shared by experts. Piggybacking on this last point, the usage of existing standards can also help expand the metadata scope to be included in the model and further elaborate on reporting. With our ontology that covers the metadata of the model card report, there is also the potential to index these reports that can assist in searching and querying. Furthermore, the ability to index can furnish the capacity to collate the information for analysis and aggregation. Combined with the expressiveness afforded through OWL2 to code ontologies, there is the potential to enrich the querying facilities and provide machine-driven inferences. In addition to describing the development of this ontology, we will outline specific use cases to point toward future direction of this work.

*With an ontology representing the documentation structure of model cards we can transform model cards to a computable artifact - one that can link information about machine learning models, leverage semantic reasoning to link the information and open up software tooling opportunities, aggregation of model card data, and querying of the information for biomedical machine learning research*. We demonstrate the application of the ontology by using two bioinformatics-related model card samples - one pertaining to a machine learning model for adverse effects of stent implantation and the other for social networks of HIV patients. Also we provide insight to our proposed software publication engine that leverages ontology-based reasoners, like FaCT++ [22], to manage the informational pieces of the model card report and allow sophisticated querying functionality. Overall the implication of this work could supplant static textual model cards reports for a machine readable version that would support FAIR-based principles and improve clinical informatics research.

## Results

The resulting ontology was encoded for OWL/XML (.owl) and Turtle syntax (.ttl) and is available at our GitHub link [23]. The current class count of the final OBO-aligned MCRO numbers at 954 with 145 object properties, 25 data properties, and 2147 logical axioms. The core foundation of MCRO was 38 classes, 11 data properties, and 79 logical axioms. Table 1 shows the breakdown of these numbers according to the imported ontologies (SWO, IAO, SKOS, PROV-O).

**Table 1:** Account of number of ontology features for the incorporated ontologies in MCRO

	Classes	Object properties	Data properties	Logical axioms
MCRO-Core	38	0	11	79
*IAO*	259	50	4	510
*PROV*	31	44	6	181
*SWO*	621	34	3	1335
*SKOS*	5	17	1	42
MCRO	954	145	25	2147

Italics indicate imported ontologies for MCRO

Essentially, an encoded ontology (in OWL 2) describes axioms (i.e. logical statements) adhering to the conceptual schema level, which in turn, formalizes other encoded axioms that describe instance data. The task of the reasoner is to ensure that axioms satisfy these logical constraints and express information that is sound. Using our earlier example, *software* and *algorithm* are two class-level concepts that are disjointed, meaning there is no conceptual overlapping of instantiated data. If we incorrectly encode an assertion *differential evolution (acme-variant)* is a *software* AND an *algorithm*, this would result in the ontology’s representational model to be logically inconsistent, and the software reasoner would indicate this inconsistency. While the example stated is simple, reasoners have a crucial role in validating the logical soundness of an ontology, particularly large ontologies with complex axioms. Considering that our work imports existing OBO ontologies, passing logical checks also ensures alignment with these imported ontologies which signals interoperability to the body of standardized biomedical ontologies.

Within the Protégé environment, there exist built-in facilities to utilize software reasoners to check the logic of the ontology. For this work, we used the FaCT++ reasoner (v1.6.5) that is generally known to accommodate various axioms types and have one of the fast performance [24]. Activating the reasoner will load the ontology, and the reasoner will preform the logic-based checks of the axioms. The Protégé environment will flag and alert the user of any unsatisfiable inconsistencies that need to be rectified in order for the ontology to pass the validation process. The resulting check with the FaCT++ reasoner (v1.6.5) evaluated the logical consistency of the final OBO-aligned MCRO, and it revealed no logical inconsistencies thereby passing the logic checks of the coded axioms.

**Fig. 1:**
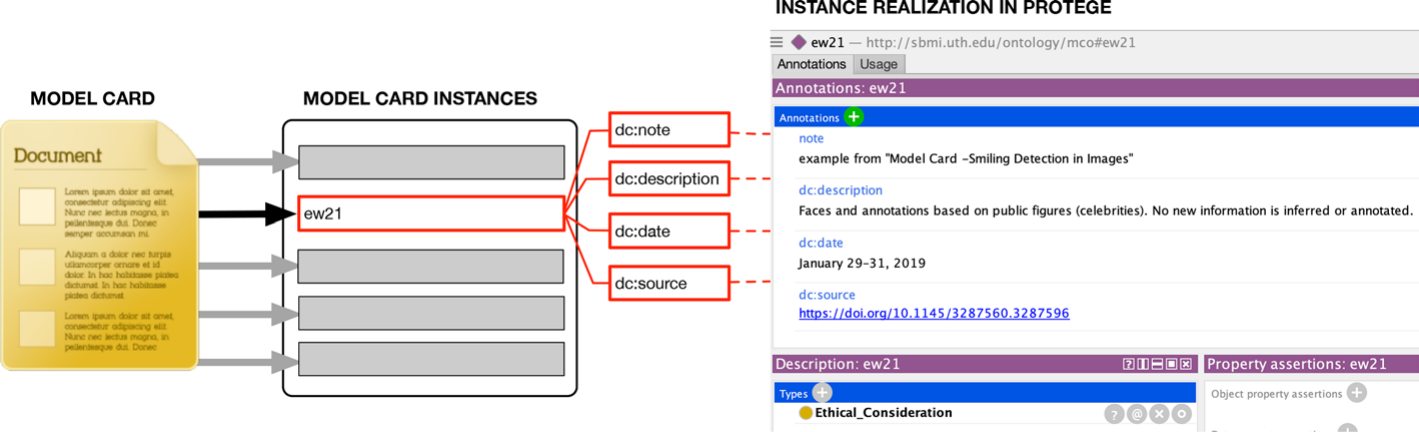
An example showing how pieces of the textual information are mapped to instances of the Model Card Report Ontology concept. “ew21” is the identifier associated with a part of the model card that has links to data annotations (Dublin Core’s *note*, *description*, *date*, *source*). In this example, we used the text from the Ethical Consideration section from the inception paper [4]

Figure 1 shows how the ontology would operationalize a sample model card report. Each textual information of a model card report would map to a concept from our ontology model. The textual information from the model card is expressed as instance data of the ontology and has metadata annotation associated with it. At our disposal, the imported SKOS and PROV-O framework provides an extensive array of annotations properties including from Dublin Core.

**Fig. 2:**
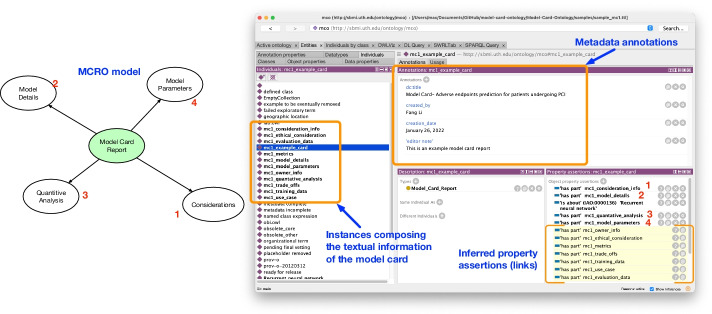
A descriptive visualization showing the instances displayed through Protégé and their mapping to a segment of the MCRO model. The numbers from MCRO model (right) demonstrates how the model is manifested in the ontology artifact with matching numbers in the Protégé viewer. The yellow highlights shows the inferred assertions resulting from the OWL2 semantics and the FaCT++ reasoner

We produced two sample model card documents pertinent to the bioinformatics domain. One titled “Adverse endpoints prediction for patients undergoing PCI” details a recurrent neural network model for prediction of adverse outcomes from coronary artery stent implantation, and the other titled “Incorporating social network information to predict HIV status” details the use of graph convolutional network to predict HIV infection from social network data. Similar to what was described in above, each textual part of the two documents were encoded into the final ontology model as instance data and aligned to their corresponding concept. To show an example, Fig. 2 displays a general view from the Protégé environment of an encoded model card report sample. Also Listing 1 shows the underlying encoding of model card instance for one of the samples in Turtle syntax.

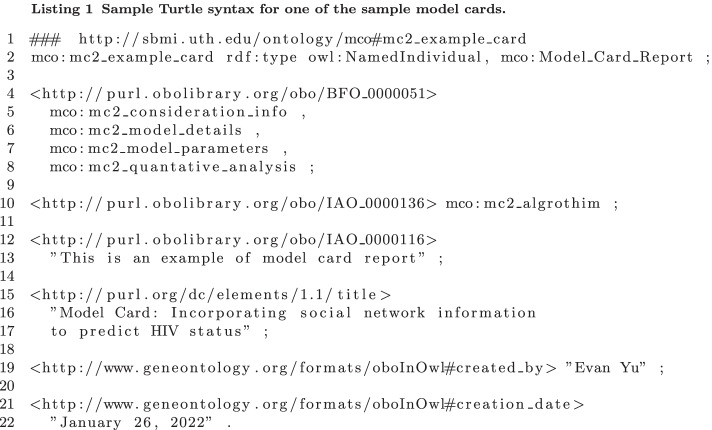



**Fig. 3:**
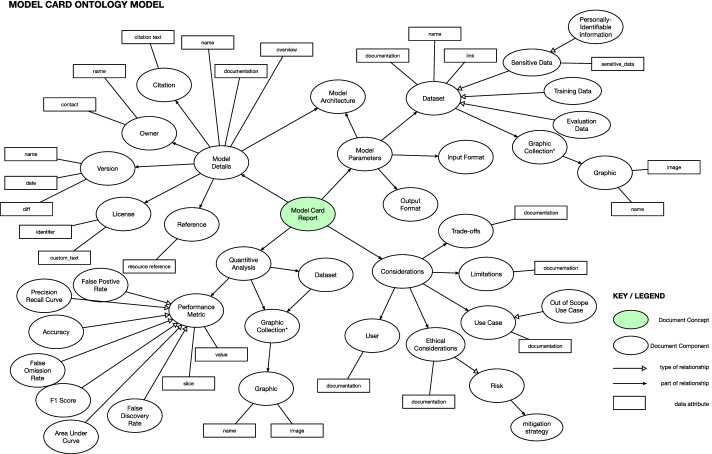
Visualization of the abstraction for the Model Card Report Ontology

Essentially, each major concept from the MCRO model (refer to Fig. 3 for details) is utilized as instance data and annotated with metadata and linked to text of the model card (see Fig. 7). Both Figs. 7 and 8 show text of the model card represented as data attributes, e.g., in Fig. 7 as *documentation* for the *Trade off* and in Fig. 8 as the *overview* attribute for *Model Details*.

Also revealed in the figures are machine-based inferences generated by the semantics of the coding. In Fig. 2, due to the transitivity of the *has part* object property (and its inverse of *part of*), the FaCT++ reasoner produces all of the instances belonging to the model card sample beyond the first degree connection. For the sample in Fig. 7, the instance for *Consideration* information content coded that it has *Trade off* content part (See Listing 2, lines 6-7). *BFO_0000051* (*has part*) is defined as transitive property with an inverse equivalent (*part of*). This produces the inferred *part of* connection seen in Fig. 7.
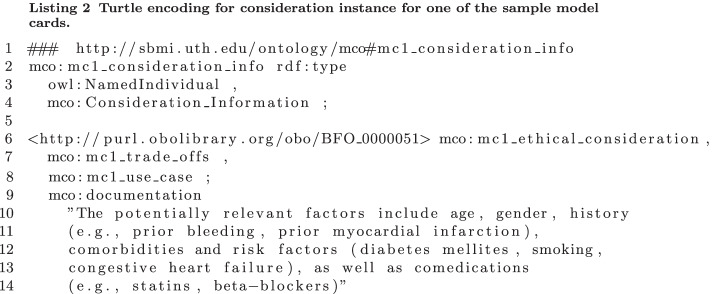


This is also evident in the example in Fig. 8. The *Model Detail* instance has content part *User* information instance. Yet in this example, because of the transitive function of *BFO_0000051*, the reasoner also infers that *User* information instance (University of Texas Health Science Center, refer to “4” of Fig. 8) is also part of the instance of *Model Detail* and the referring model card. Both samples are available at the our GitHub repository to illustrate the overall coverage of the ontology model and the ability to translate document model cards to a computational format.

## Discussion

Model card reports are one tool suggested by the National Institute of Health for their Bridge2AI initiative to advance behavioral and biological sciences impacted by research utilizing machine learning models. In this paper, we discussed our effort in developing the Model Card Report Ontology to represent and standardize the information in a computable format to allow the reports to be machine-readable. This work is the first effort to develop an ontology model toward making model cards reports computable and serve scientific endeavors of FAIR for biomedical machine learning models.

To enforce some rigorous standard to our model, we utilized existing ontology resources from the OBO Foundry that adheres to strict principles for standardizing their ontology models. The benefit of using these community-agreed standards is the semantic precision that can be leveraged to evoke meaningful data and information. For example, we heavily used the partonomy relationship of *has part*. Partonomy relationships between concepts have varied and nuanced interpretations. A quick glance of this type of property [25] provide over 10 types that express very granular and nuanced meaning. Likewise, the same can be said of the entity categories that many bio-ontologies are aligned with in the BFO framework. Overall as noted by Obrst and colleagues [26], the more semantically rich the ontology, the more useful the artifact will be for sophisticated tools and applications. This is in effect would help formalize model card reports for application consumption and distribution.

Using our current version of the Model Card Report Ontology, we were able to transform the model card samples into ontology-based format. An issue with model card reports is that they are static documents. With an ontology-based representation we provide machine-readable artifact that can link to heterogeneous digital resources using a composition of formal ontologies. Also as ontology-based representational artifact we provide a formal structure for consistency and manage to derive coverage for the model card report.

While the ontology manages to cover structure and concepts of the model card report, we were limited by the incorporated ontologies. For example, specific types of neural network algorithms were not available from SWO, so we relied on the “generic” *neural network* for our sample. One of the benefits of using ontologies to encode model card reports is the possibility to further extend the representation with additional ontologies. While the OBO Foundry ontologies we used were sufficient to cover the model card report there could be other ontologies, like the Population Ontology or the Ontology for Biomedical Investigations [27] that can further enrich the model. Lastly, our two samples were limited to bioinformatics-focused model card reports, and therefore, other non-bioinformatics model card reports may have unique coverage requirements beyond the sufficient coverage we presented.

From an application and tooling perspective, the ontology can be harnessed by software application interface since the ontology, in of itself, is reusable software code that serves as a computable knowledge base. The work detailed, specifically the inferencing was localized in the Protégé authoring environment to show the potential feasibility of the ontology. Software APIs for the semantic web applications, like OWL-API [28], Jena [29], HermiT API [30], etc. enables the development of tailored software applications to utilize an ontology for useful real world purposes such as machine reasoning tasks outside of the Protégé environment.

For the sample model cards that we encoded into the ontology, the instance data, the instances’ links, and their annotations were a manual process performed through Protégé. While Protégé provided a setting to experiment our approach, an ideal scenario is to have automated tools that will hide the arduous task of encoding the model card manually where the author of the model card just provides the textual content and content will link automatically using the MCRO. One proposed tool we are currently developing relates to a MCRO-driven software application that can capture the textual inputs of the model card report from an author (see Fig. 9). The author identifies the concept-related section associated with their inputted text and the system will generate an output in the form of an ontology-based artifact (e.g., RDF, JSON-LD [31], nanopublication [32]). This export file can be distributed and shared with all of the necessary alignment with the ontology schema embedded in the export. Multiple model cards of this format that have the shared schema could be queried in aggregate for analysis or service some application tool.

So in addition, another future direction is to develop the natural language processing (NLP) pipeline that will align unstructured text from scientific literature and populate/link to the ontology with assistance of the publication engine. Figure 9 alludes to an natural language processing pipeline to extract unstructured text from published research involving ML models and linking to MCRO scaffold model. In this subsystem depicted in the figure, an automated method to assist in the authoring of ontology-based model card would involve NLP methods—named entity recognition, information and knowledge extraction, controlled natural language - to extract data from unstructured document sources (research publications, existing model card, web pages, etc.). For example, named entity recognition could identify concepts or phrases that match concepts in MCRO, or information extraction methods to parse subject-verb-object data from the text that can map to the ontology’s concept level triples. Afterwards, the subsystem populates MCRO with the extracted data as instances that is coordinated by the publishing engine.

Another example of an applied use case is to utilize MCRO as an index component resource to support search services for querying model card documents and possibly aggregating their textual data and metadata for analysis. Anticipating the massive generation of datasets and tools resulting from AI and ML for biomedicine, this could assist potential biomedical and behavioral researchers toward the aforementioned goals.

### Comparison to related works

There exist some related ontology-driven work towards adjacent goals but with a focus on niche areas (annotating the development of ML or a focus on neural nets). For example, Naja, et al. developed a duo of ontologies, The System Accountability Ontology (SAO) and Realising Accountable Intelligent Systems (RAInS) ontology, for auditing AI systems to assist in evaluating the risks of ML models [33]. Their ontologies annotate the development stages (i.e. software development lifecycle) for ML-based AI systems. Their work includes a proof of concept software application to show the utilization of their ontology, essentially an authoring tool. The FAIRnets Ontology defines the layers and aspects of neural networks for the purpose of accountability [34]. This ontology is limited to just neural networks with much of the classes (69) devoted to the types of layers. The resources are available for open source and we reviewed them for comparison purposes [35–37].

Our review of the resources highlighted some unique features of our work. One is we designed and encoded a semantic representation of a model card report that is computable and standardized through the use of community generated ontology models from biomedical ontologists. While some of these related studies relied on some existing standard resources like PROV-O and Dublin Core, they do not leverage the existing available ontologies that span over the relevant domains, like software (via SWO) or IAO which helped to structure the development our model to represent model cards. In addition, without standard models there is a risk of generating siloed resources that do not have an agreed consensus on the semantics of the concepts. This also enables our MCRO to continually evolve with emerging or other existing ontologies like the Ontology of Biomedical Investigation (OBI) or any other OBO Foundry ontology that are bioinformatics-related. Also the aforementioned resources are essentially used as knowledge graphs and do not leverage any semantic features of OWL2 that could enhance the application of their ontology models. Lastly, because our work utilizes existing standard resources ours is more comprehensive and is not focused on a specific niche.

## Conclusion

The utilization of ML has advanced biomedical research in the last decade, contributing to the mass volume of complex data and tools. This has encouraged the National Institutes of Health’s interest in making tools and data more FAIR and transparent for biomedical and behavioral researchers. We introduce the Model Card Report Ontology, a representational artifact encoded in OWL2 that can contribute toward FAIR data standards and transparency of machine learning models in biomedicine research. This initial version of the ontology can enable the linking of standardized metadata and encourage the development of tools and resources to search and aggregate machine learning models for health researchers. With two general bioinformatics-related model cards we were able to show how the model cards would translate to an ontology-based artifact and how it can rely on machine-based inferences to link text indexing and search. Future work will involve developing an engine that harnesses the ontology for application use, and continually improving the conceptual scope of the ontology to accommodate unique model card reports.

## Methods

**Fig. 4:**
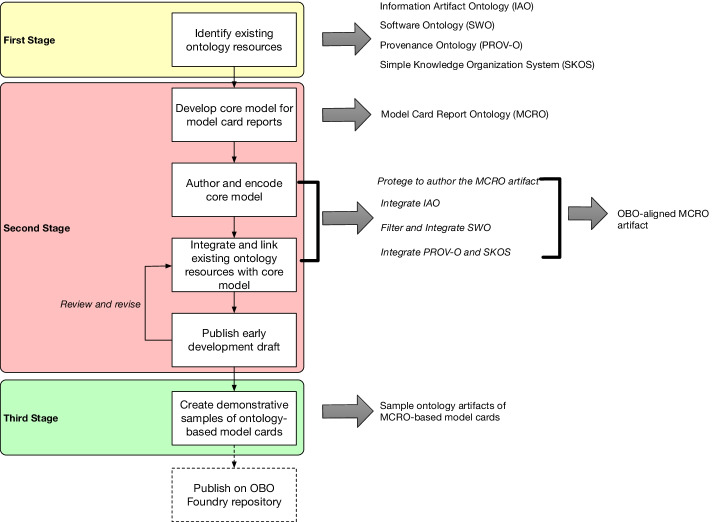
Outline of the process in developing the Model Card Report Ontology

The basic steps in constructing the Model Card Report Ontology (MCRO) involved 1) identifying existing ontological resources that can be leveraged by MCRO, 2) developing the core framework of MCRO to synthesize the heterogeneous resources and fill any missing gaps to cover the scope of the model card report, and 3) testing the application of the ontology to link model card information and how integrative software usage. In Fig. 4, we outline the process in the development of the Model Card Report Ontology starting with identifying ontologies from the OBO Foundry that coincide with concepts with the domain, and accommodating conceptual gaps that are not supported by the existing ontology models by drafting a core abstraction with collaborators. Later stages involve encoding the core model and linking the core model with the identified existing ontologies. Our eventual goal is to publish this ontology toward making this a formal standard that aligns with other biomedical and health ontologies for reuse and sharing.

### Integrating OBO foundry ontologies

The stated goal of the OBO Foundry is to “develop a family of interoperable ontologies that are both logically well-formed and scientifically accurate” [9]. Following a prescribed approach to building an ontology [38], we leveraged existing OBO Foundry ontologies to enhance the terminological scope and also to promote reusability of existing ontology-based sources. Extending our core model to these ontologies will help align our work with a common architecture shared with a variety of consistent biomedical and life sciences ontologies.

**Fig. 5:**
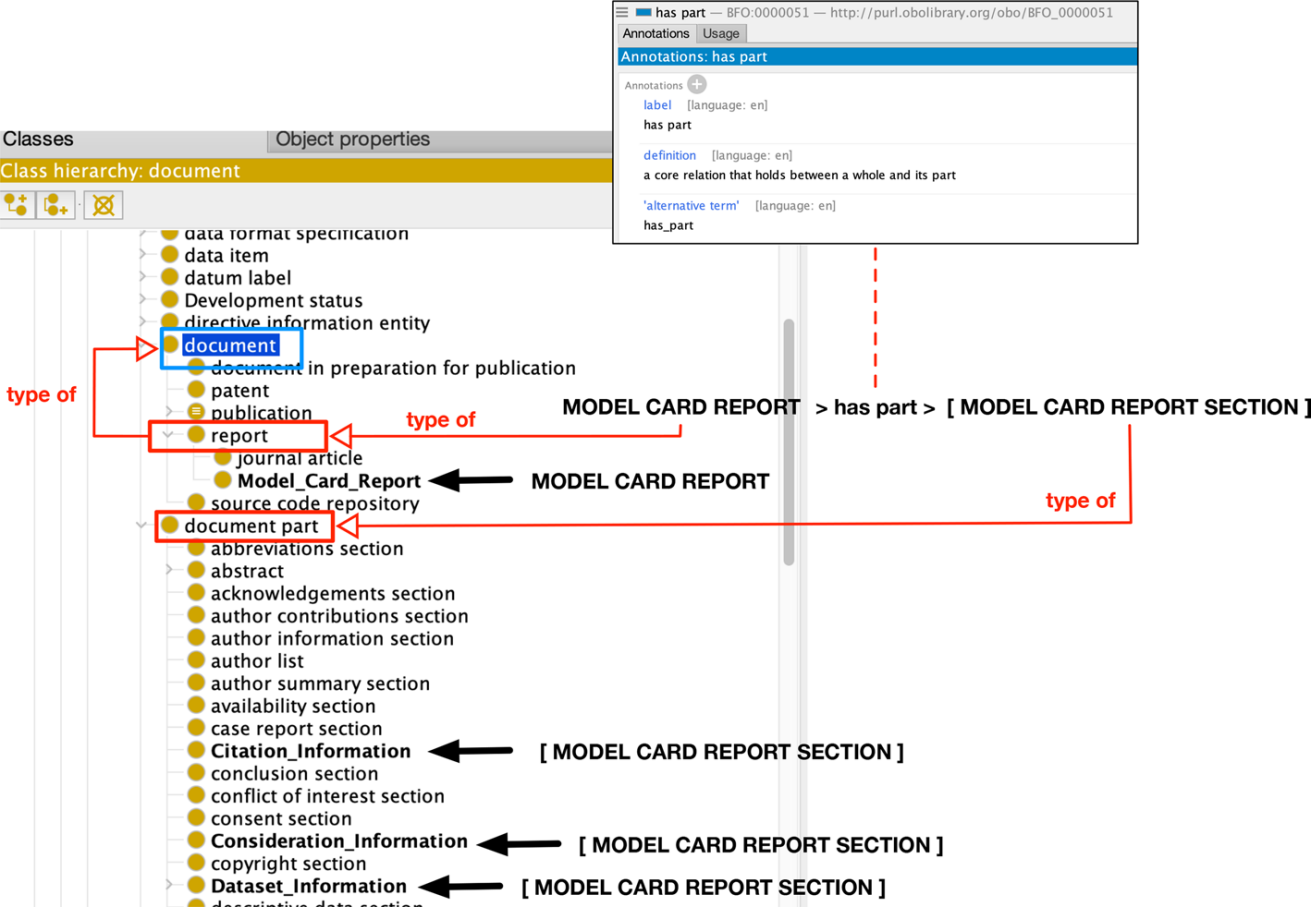
Annotated Protégé screenshots of how the Information Artifact Ontology concepts of *report*, *document part*, and *has part* are aligned to the Model Card Report Ontology concepts (Model Card Report and its model card sections of Citation, Consideration, and Dataset Information)

We relied primarily on the Information Artifact Ontology (IAO), which represents a myriad of high-level information entities. Basically, a model card report is a report [4], which aligns to IAO’s *report* concept (which is a type of *information content entity*). Each section of the model card is viewed as part of the document (i.e., IAO’s *document part*) that composes the sectional and textual pieces of the model card. These sections are tied together using IAO’s *has part* property (*BFO_0000051*), which defines a specific relationship in an ontology. Figure 5 outlines MCO’s concept connection with the main classes of the IAO.

An important domain involved in machine learning models are software and periphery concepts involving software—application of the software, metadata specific to software, algorithms, etc. In addition to IAO, we utilized concepts and terminologies from the Software Ontology (SWO), particularlily, concepts such as *data*, *license*, *version*, *algorithm*, etc. These software concepts relate to the various concepts described in our core model to be discussed in the next subsection, and provide exhaustive list of software-related terminologies that can be integrated to our ontology model.

**Fig. 6:**
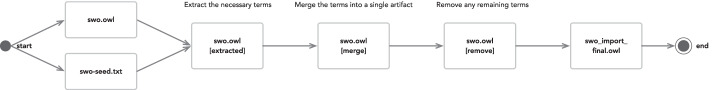
An outline of the process of to generate a subset of the Software Ontology to import into the Model Card Report Ontology

Since SWO relies on IAO, and to avoid redundancy, we extracted a subset SWO that covers target terms we need for model card. We used OBO’s ROBOT to extract these essential concepts. ROBOT is a command line software tool that supports a variety of common development tasks when working with ontology files [39]. From SWO (swo.owl), we needed to extract the targeted terms (swo-seed.txt) and export them to a separate filtered version of SWO (swo_import_final.owl). Figure 6 shows the basic steps starting with the target seed terms and the SWO, followed by extraction and merging of the terms to its own ontology file. The resulting subset of SWO was uploaded to GitHub and then imported to furnish the MCRO model.

**Fig. 7:**
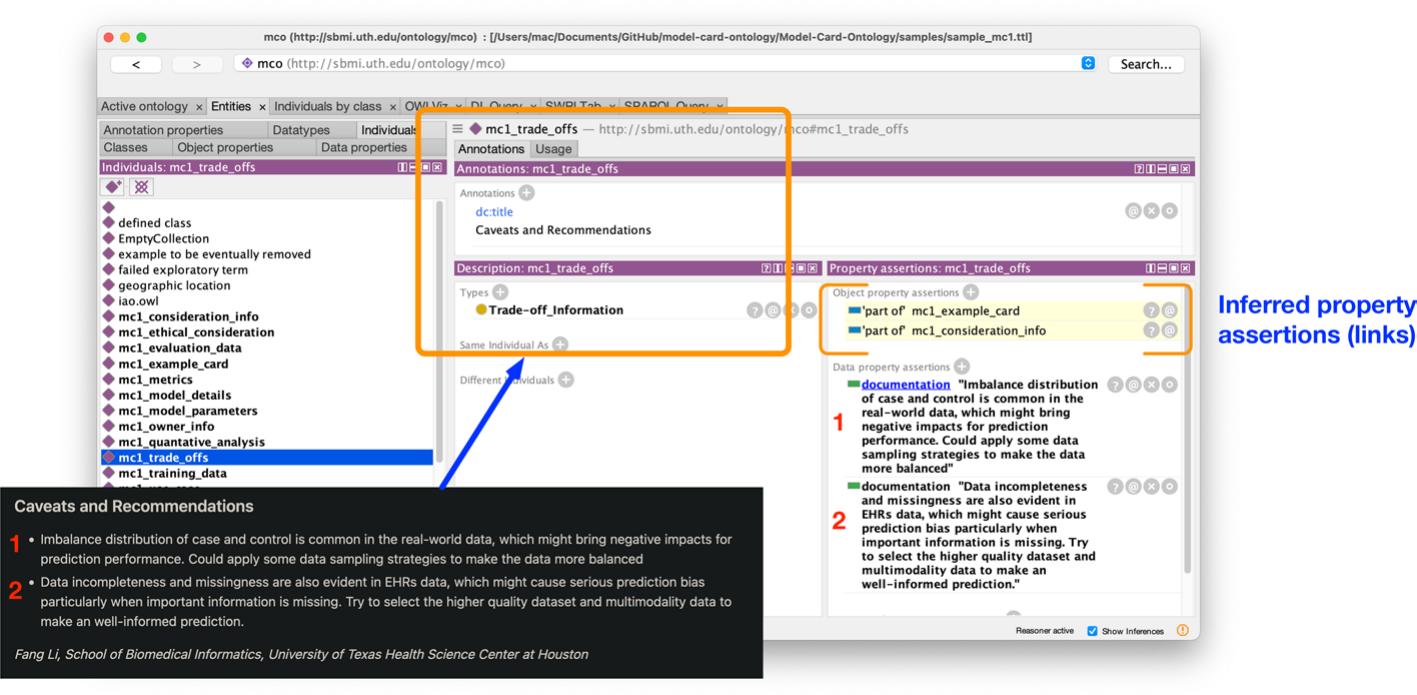
An example of some of the text from the sample model cards (*Trade off* information) mapped to the instances and the inferences generated in the Protégé environment (highlighted in yellow)

**Fig. 8:**
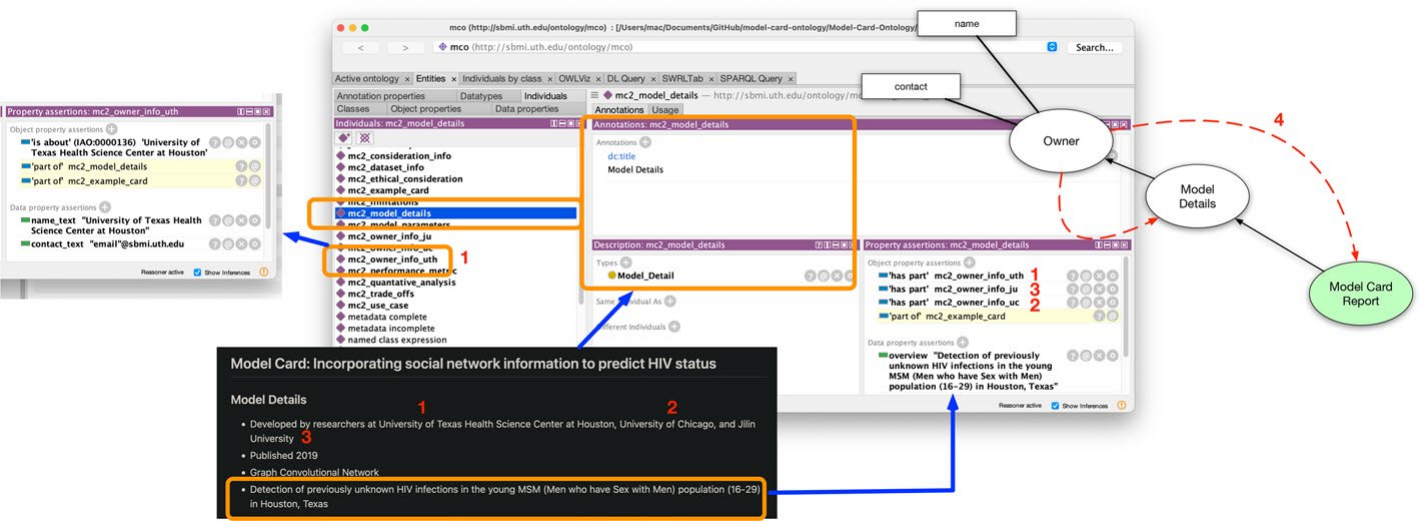
An extensive visualization of the mapping of sample text as instance data of the Model Card Report Ontology, including displaying the transitive inference of one of the object properties. The dotted lines in red shows the inference generated from “4” which is an inference

**Fig. 9:**
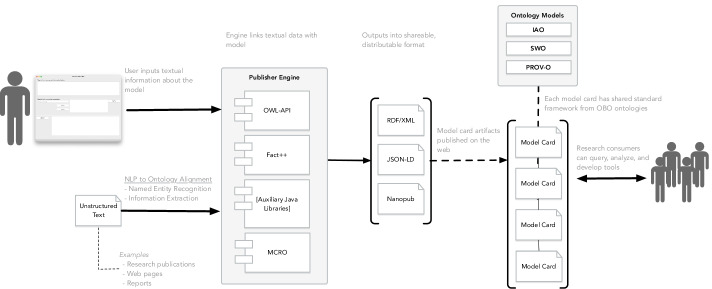
An applied use-case of the Model Card Report Ontology for software publishing engine to generate ontology-based model card artifacts as RDF or JSON. This applied use case involves a manual authoring method or a proposed automated natural language processing (NLP) method

The Provenance Ontology is another OBO Foundry ontology that contains terminologies to annotate the origination of data and metadata . This ontology assists in providing coverage for authorship and metadata of resources, like a machine learning model resource (citation, dates, etc.). Lastly, we incorporated the Simple Knowledge Organization System (SKOS) ontology [40] for facilities to support annotations of similar concepts, alternative labels, etc. In the next subsection we discuss how these open ontologies are integrated to the core model.

### Development of the core of model card report ontology

We examined the inception paper by [4] and the source code files for generating model card documents [41]. From our review of the sources, we drafted an abstraction that approximated the structure of the model card report to generate our ontology. The core concept of this representation is the *Model Card Report* concept which branches onto four main sections -* Model Parameters* (describes the assembly of the model, like algorithm, datasets for training and evaluation, the data format for input and output, etc.), *Considerations* (describes the limits, caveats, and impact on intended users), *Model Details* (general basic information of the model, like licensing, version, developers and owners, name, etc.), and *Quantitative Analysis* (describes the detailed data of model’s performance). Essentially, the *Model Card Report* concept represents one model card report and its related textual sections. Figure 3 shows an overview of the representation of the abstraction.

Using the representation, we authored the Model Card Report Ontology using Protégé [42]. The Model Card Report Ontology relied on imports of the aforementioned ontologies discussed in *Integrating OBO Foundry Ontologies*. Alluded earlier, model cards are a type of report and the *Model Card Report* concept was subclassed as an IAO’s *report* concept. The concept *Graphic Collection* that groups figures of quantitative data is sub-classed as SKOS’s *Collection* concept. Most of the links between concepts described in Fig. 3, utilizes BFO and IAO’s *has part* of link the various information sections of the model card and *is about* was used to relate the model card card sections to other concepts found in SWO and IAO. Some of the concepts used alternative terms to describe certain sections, e.g., *Caveats* instead of *Trade offs*. This was facilitated with the *alternative label* annotation. Additional links and resources from the OBO Foundry ontologies are described in Table 2.

**Table 2:** Integration of OBO Concepts with Model Card concepts encoded in the ontology

Model card concept	OBO property link (URI)	OBO concept (URI)
Data set	Is about ($\star$)	Data set (http://purl.obolibrary.org/obo/IAO_0000100)
Model architecture	Is about ($\star$)	Algorithm (http://purl.obolibrary.org/obo/IAO_0000064)
Format information	Is about ($\star$)	Data format specification (http://purl.obolibrary.org/obo/IAO_0000098)
Version information	Is about ($\star$)	Version number (http://purl.obolibrary.org/obo/IAO_0000129)
Reference information	Equivalent to (${\mp }$)	Reference section (http://purl.obolibrary.org/obo/IAO_0000320)
Graphic	Equivalent to (${\mp }$)	Graph (http://purl.obolibrary.org/obo/IAO_0000038)
Citation information	Has part ($*$)	Citation (http://purl.obolibrary.org/obo/IAO_0000301)
License information	Is about ($\star$)	License (http://www.ebi.ac.uk/swo/SWO_0000002)

$\star$ http://purl.obofoundry.org/obo/IAO_0000136, ${\mp }$http://www.w3.org/2002/07/owl#owl:equivalentClass, and $*$http://purl.obolibrary.org/obo/BFO_0000051

### Demonstration use case for MCRO

To show proof of concept of this work, two bioinformatics researchers who are primary investigators on experimental machine learning endeavors, specifically for ML models on electronic health records (EHR) data to classify and predict adverse effects of stent operations and on social network survey data for HIV transmission among young MSM (Men who have sex with Men). We reviewed the format of the model card report and produced two sample model cards for the two experimental ML-based bioinformatics research projects. With the finalized MCRO, we authored two ontology-based equivalent using MCRO as the scaffold conceptual schema. Both the textual form and the ontology artifact form are available at the GitHub repository [23].

## Data Availability

Model Card Report Ontology: https://github.com/UTHealth-Ontology/MCRO.

## Abbreviations

FAIR: Findable, Accessible, Interoperable, and Reuse
NIH: National Institute of Health
AI: Artificial intelligence
ML: Machine learning
FDA: Food and Drug Administration
OBO: Open Biological and Biomedical Ontology
BFO: Basic Formal Ontology
OWL2: Web Ontology Language
RDF: Resource Description Framework
MCRO: Model Card Report Ontology
IAO: Information Artifact Ontology
PROV-O: Provenance Ontology
SWO: Software Ontology
SKOS: Simple Knowledge Organization System
EHR: Electronic health record
MSM: Men who have sex with men
NLP: Natural language processing
SAO: System Accountability Ontology
RAInS: Realising Accountable Intelligent Systems
OBI: Ontology of Biomedical Investigation
URI: Uniform resource identifier
